# Major adverse cardiovascular events’ reduction and their association with glucose‐lowering medications and glycemic control among patients with type 2 diabetes: A retrospective cohort study using electronic health records

**DOI:** 10.1111/1753-0407.13604

**Published:** 2024-10-21

**Authors:** Haowen Hsu, Paul Thomas Kocis, Ariana Pichardo‐Lowden, Wenke Hwang

**Affiliations:** ^1^ Department of Clinical Pharmacy School of Pharmacy, College of Pharmacy, Taipei Medical University Taipei Taiwan; ^2^ Department of Public Health Sciences College of Medicine, Penn State University Hershey Pennsylvania USA; ^3^ Department of Pharmacy Penn State Health Milton S. Hershey Medical Center Hershey Pennsylvania USA; ^4^ Department of Pharmacology College of Medicine, Penn State University Hershey Pennsylvania USA; ^5^ Department of Medicine Penn State Health Milton S Hershey Medical Center Hershey Pennsylvania USA

**Keywords:** glucose‐lowering medications, glycemic control, major adverse cardiovascular events, primary prevention, type 2 diabetes

## Abstract

**Background:**

Cardiovascular diseases are a common cause of death among patients with type 2 diabetes (T2DM). Major adverse cardiovascular event (MACE) risks can be significantly reduced under adequate glycemic control (GC). This study aims to identify factors that influence MACE risk among patients with T2DM, including Hemoglobin A1c variability score (HVS) and early use of MACE‐preventive glucose‐lowering medications (GLMs).

**Methods:**

We conducted a longitudinal cohort study to retrospectively review electronic health records between 2011 and 2022. Patients with T2DM ≥18 years without previous stroke or acute myocardial infarction (AMI) were included. Cox regression was utilized to investigate MACE risk factors and compare MACE risk reduction associated with early use of MACE‐preventive GLMs.

**Results:**

A total of 19 685 subjects were included, with 5431 having MACE, including 4453 strokes, 977 AMI, and 1 death. There were 11 123 subjects with good baseline GC. Subjects with good baseline GC had 0.837 (confidence interval [CI]: 0.782–0.895) times lower MACE risk than their counterpart. Subjects with a single MACE‐preventive GLM at baseline with continuous use >365 days showed a decreased MACE hazard ratio (0.681; CI: 0.635–0.731). Among all MACE‐preventive GLMs, semaglutide provided a more significant MACE‐preventive effect.

**Conclusions:**

This study identified that GLM, early GC, and HVS are MACE determinants among patients with T2DM. Novel GLM, adequate GC, and reduction of HVS can benefit MACE outcomes.

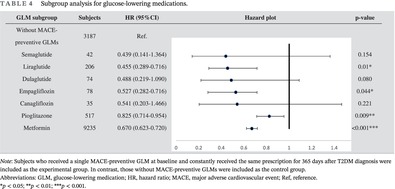

## Graphical abstract

Highlights

- Good baseline glycemic control (GC) can reduce the risk of future major adverse cardiovascular events (MACEs) by 16.3%.
- MACE risk increases as the Hemoglobin A1c variability score (HVS) increases, and the HVS boundaries differ between good GC and poor baseline GC.
- Subjects with MACE‐preventive glucose‐lowering medications (GLMs) at baseline had 0.681 times lower MACE risk.
- Among all MACE‐preventive GLMs, semaglutide potentially had the higher MACE‐preventive effect (hazard ratio [HR]: 0.439; CI: 0.141–1.364), while pioglitazone (HR: 0.825; CI: 0.714–0.954) had the least in relative.

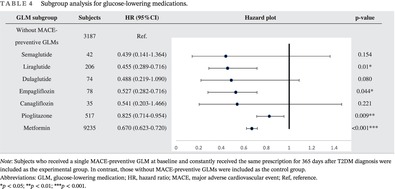

## 1 INTRODUCTION

Diabetes mellitus is a chronic disease^1^ characterized by hyperglycemia due to insulin deficiency and resistance.^2^ Type 1 diabetes (T1DM) results from cell‐mediated autoimmune pancreatic beta‐cell destruction.^2^, ^3^ The causes of type 2 diabetes (T2DM) are often multifactorial and may involve the liver, β cells, kidney, brain, and more,^2^, ^4^ accounting for 90%–95% of diabetes cases.^2^, ^3^ Globally, the prevalence of T2DM increased from 462 million in 2017 to 537 million in 2021,^5^ and it is estimated to increase from 643 to 783 million in 2030 and 2045,^6^ respectively, thereby posing tremendous global public health threats regardless of the country's level of development.^7^ While T2DM impacted regions and countries differently,^7^ T2DM rose to the top 10 global leading causes of death in 2017, compared with the 14th place in 2007.^8^ In the United States, despite the fact that the incidence of diagnosed diabetes among adults ≥18 years started to decrease since 2009, the prevalence is still rising.^9^ An increasing prevalence and decreasing mortality revealed the significance of diabetes‐related complication prevention since complications may develop over time.^9^, ^10^ As a consequence, diabetes has greatly impacted the U.S. economy and is listed as the most expensive chronic illness,^11^, ^12^ with $237 billion spent on direct medical costs and $90 billion on indirect costs yearly.^12^

Diabetes dramatically impacts health outcomes, which was emphasized by a study reporting that three out of four patients with T2DM had at least one comorbidity present by the time of diagnosis, and 44% had at least two.^13^ T2DM affects the circulatory, digestive, skeletal muscle, nervous, and immune systems.^14^, ^15^ Among the T2DM complications, macrovascular complications, including coronary artery disease (CAD), stroke, and other cardiovascular diseases (CVD),^16^, ^17^, ^18^ are common causes of death among patients with T2DM in the United States.^19^, ^20^ Heart disease and stroke occupied the first and fifth leading causes of death in 2021, respectively.^21^ To effectively prevent patients with T2DM from developing CVD, multiple modifiable risk factors (e.g., glycemic control [GC], smoking, obesity, and physical activities)^22^, ^23^, ^24^ should be addressed, and nonmodifiable risk factors^22^, ^24^ need to be concurrently considered. Well‐established evidence supports that GC can help prevent microvascular and macrovascular complications.^24^, ^25^, ^26^ In addition, failure to maintain stable Hemoglobin A1c (HbA1c) with a higher HbA1c variability score (HVS) may contribute to an increased risk of major adverse cardiovascular events (MACEs) due to hypoglycemia episodes and other acute short‐term T2DM complications.^27^ Among all glucose‐lowering medications (GLMs), some have been suggested by the American Diabetes Association (ADA) for their proven benefits in MACE prevention, including the following^28^:

- Biguanide
  - metformin
- Glucagon‐like peptide‐1 receptor agonists (GLP‐1 RAs)
  - dulaglutide, liraglutide, and semaglutide
- Sodium‐glucose transport 2 (SGLT‐2) inhibitors
  - canagliflozin and empagliflozin
- Thiazolidinediones
  - pioglitazone

This study aims to (1) identify MACE risk factors among patients with T2DM and (2) study the influence of GC and HVS on MACE risk. Moreover, (3) this study will also discuss the early use of MACE‐preventive GLMs, defined as GLMs that can reduce MACE incidence, in primary MACE prevention among patients with T2DM.

## 2 STUDY DESIGN AND METHODS

We conducted a longitudinal cohort study to retrospectively review electronic health records (EHRs) from the Penn State Health and the Penn State College of Medicine. Penn State is part of the PaTH Clinical Research Network, supported by the Patient‐Centered Outcomes Research Institute and Patient‐Centered Clinical Research Network (or PCORnet).^29^, ^30^ Updated quarterly, PaTH contains deidentified EHR data from over 1 million patients who received care at the Penn State Health System, including five hospitals and over 100 practice locations since January 1, 2011. This study utilized retrospective data between January 1, 2011, and August 30, 2022. With up to 11 years of surveillance, the subject's baseline and follow‐up periods were distinguished by their cohort entry event (Figure S1). The Human Subjects Protection Office of the Penn State University Institutional Review Board approved the study.

### 2.1 Study population and cohort definition

The study team constructed a rule‐based cohort with a rigorous data selection process, including four configurations (Figure S2): cohort entry event, inclusion criteria, qualifying cohort, and cohort exit event based on the Observational Health Data Science and Informatics (OHDSI.org) guidelines.^31^

#### 2.1.1 Cohort entry event

This study defined the cohort entry event as a T2DM diagnosis.^31^ The study team developed a three‐stage subject selection process (Figure S3) to reference T2DM diagnosis. The three stages were (1) whether the patient had an ICD‐9 (250.X0 and 250.X2)^32^, ^33^, ^34^ or ICD‐10 code (E11.X)^33^, ^34^ for T2DM; (2) if any GLMs in the medication record other than metformin; and (3) if had metformin only, whether presenting ≥2 elevated HbA1c values, defined as HbA1c >7%,^35^ or one elevated HbA1c plus one random blood glucose ≥180 mg/dL. Three subject selection stages came in a particular sequence. If patients failed to be captured by the former stage, the following stage would be used. ICD‐9 (250.X1 and 250.X3)^32^, ^33^, ^34^ and ICD‐10 (E10.X)^33^, ^34^ for T1DM were applied to exclude those captured by stages 2 and 3 in case GLP‐1 RAs, SGLT‐2 inhibitors, and metformin may have been prescribed for T1DM patients for their non‐GC‐control benefits.^36^, ^37^, ^38^ Once subjects were selected, their index date was the earliest T2DM diagnosis or GLM prescription date. The development of the T2DM diagnosis process was referenced from the SUPREME‐DM project^39^ and by Upadhyaya et al.^40^

#### 2.1.2 Inclusion and exclusion criteria

Three inclusion criteria refined the initial event cohort: (1) age ≥ 18 at baseline, (2) received at least one GLM in the prescription records during the investigation window, and (3) without a past medical history of a stroke and/or acute myocardial infarction (AMI) diagnosis during the look‐back period.

#### 2.1.3 Qualifying cohorts

To be qualified for the final study cohort, subjects should have ≥1 HbA1c result within their first T2DM year and ≥3 HbA1c results during the follow‐up period. Furthermore, subjects should have a patient encounter record >365 days after T2DM diagnosis. Those without encounter records >365 days were considered incident cases,^41^ revealing insufficient longitudinal information for our study. Additionally, <365 days of encounter data indicated the possibility of a one‐time visit without regular follow‐up. This can be understood as having a higher probability of data discontinuity and missing data and decreasing the study's reliability.^42^ We excluded subjects who only had emergency department (ED) visits and/or inpatient stays ≥365 days from the index date and only included outpatient visits to capture subjects with regular follow‐ups. Along with other criteria used in cohort selection, the study team constructed a loyalty cohort to minimize the data missingness and bias in the EHR database and provide a meaningful cohort.^43^

#### 2.1.4 Cohort exit event

The end of the follow‐up was addressed by the events below. (1) MACE observed: since MACE was the event we intended to observe, subjects exited the cohort once MACE was observed; (2) disenrollment: reasons for disenrollment could be many, such as moving to another region or state and transitioning to a different healthcare system outside the current EHR system; (3) right censoring: MACE was not observed before the last available encounter or before the study cutoff date (August 30, 2022).

### 2.2 Baseline characteristics

Data from the final study cohort were extracted from the Penn State PaTH to Health datasets. Sex, race, ethnicity, smoking status, body mass index (BMI) (underweight: BMI < 18.5 kg/m^2^; normal: 18.5–24.9 kg/m^2^; overweight: 25–29.9 kg/m^2^; obese: 30–39.9 kg/m^2^; severely obese: BMI ≥40 kg/m^2^),^44^ and years of follow‐up were included as demographic information. The subject's baseline age was calculated by reducing the T2DM identification date by birth date. The difference between cohort entry and exit date calculated years of follow‐up. Smoking status and BMI were the subjects' earliest records after T2DM diagnosis. In addition to the variables above, baseline clinical information and medications, such as comorbidities, complications, HbA1c, and GLMs, were also considered. Comorbidities, complications, and GLMs prescription records within 90 days after T2DM identification were included. Complications were identified using T2DM's ICD‐9/10 codes.^32^, ^33^, ^34^ We utilized a 17‐item Charlson comorbidity index (CCI)^45^ to describe patients' baseline comorbidities. The severity grade was referenced from Huang et al., with 1–2 CCI scores as mild, 3–4 CCI as moderate, and ≥5 CCI as severe.^46^ The mean of the first T2DM year systolic blood pressure (SBP) and diastolic blood pressure (DBP) assessed their baseline SBP and DBP control. The earliest low‐density lipoprotein (LDL) result after T2DM diagnosis assessed their baseline LDL control. Poor SBP and DBP control were defined as mean SBP >130 mmHg and mean DBP >80 mmHg, respectively.^47^, ^48^ Poor LDL control was defined as LDL >130 mg/dL.^48^, ^49^ Those with missing baseline characteristics were classified as “unidentified” in each covariate. In this study, GLMs included injectable and oral dosage forms (Table S1).

Baseline GC was calculated as the mean of all HbA1c results of the subject's first T2DM year. The study team used HVS to assess glucose variability as HVS is more translatable than traditional measurements (e.g., standard deviation [SD] and percent coefficient of variation) in the clinical base,^50^ which provides a more practical approach for healthcare providers. HVS was calculated as the percentage of HbA1c fluctuation (increase or decrease by ≥0.5% compared with the former HbA1c result) and then multiplied by 100.^50^ For instance, the sequence of HbA1c, 5.6%, 6.4%, 6.0%, and 7.0% provides a score of 66.67 (fluctuation: +0.8%, −0.4%, and + 1.0%; three in total and two increase or decrease by ≥0.5%). Subjects were divided into five HVS sectors (0–20, 21–40, 41–60, 61–80, and 81–100). The glycemic goal was modified based on the patient's age. Based on updated ADA recommendations, the adequate glycemic goal is <7% for those <65 years old and <7.5% for older adults ≥65 years old.^35^, ^51^ HbA1c values exceeding the age‐modified goal would be identified as elevated.

### 2.3 Study outcome

According to a systematic review, stroke and AMI were the most common MACE components in observational studies, with stroke, AMI, and all‐cause death being the most observed combination.^52^ Therefore, three MACE components were selected in this study: (1) stroke (ICD‐9 in 433.X and 434.X^52^, ^53^, ^54^, ^55^; ICD‐10 in I63.X^53^, ^55^, ^56^, ^57^), (2) AMI (ICD‐9 in 410.X and ICD‐10 in I21.X and I22.X),^52^, ^58^, ^59^ and (3) all‐cause death.

### 2.4 Statistical analysis

Descriptive statistics explicitly presented cohort demographics, with mean ± SD and percentages for expressing continuous and categorical variables. Kaplan–Meier (KM) survival analysis and univariate and multivariate Cox regressions were used to evaluate determinants of MACE incidence among patients with T2DM. The KM survival analysis visualized the cumulative hazard plots of each variable. Furthermore, pairwise log‐rank tests compared the survival differences within each variable group. Univariate COX regression evaluated how each variable affected MACE risk independently. Multivariate Cox regression was constructed to adjust covariates. Multivariate Cox regression was also used for GLM subgroup analysis to investigate the relative MACE‐preventive effects of GLMs. Subjects who received a single MACE‐preventive GLM at baseline and constantly received the same prescription for 365 days after T2DM diagnosis were included and compared with those without MACE‐preventive GLMs. In another subgroup analysis, we selected those with poor baseline GC to assess the early use of novel GLMs for MACE reduction. Three subgroups in the analysis were those with MACE‐preventive GLMs at baseline, those without MACE‐preventive GLMs at baseline but later prescribed in the follow‐up period, and those without MACE‐preventive GLMs at baseline and in the follow‐up period. A GC‐stratified analysis was performed to investigate the correlation between baseline GC and HVS on MACE reduction. The hazard ratio (HR) with 95% confidence intervals (CIs) was calculated to describe the MACE risk. In this study, a *p* value <0.05 indicated statistical significance. Statistical analysis was performed with SAS (version 9.4; SAS Institute Inc., Cary, NC) and SPSS (version 28.0 for Windows, IBM Corp., NY).

## 3 RESULTS

### 3.1 Subjects selection

As of August 30, 2022, there were 1 254 012 subjects in the Penn State PaTH to Health EHR database, and 99 652 were identified as patients with T2DM (Figure S2). After being screened by three inclusion criteria, 76 418 patients with T2DM entered the qualifying cohort. There were 54 435 patients excluded from the qualifying cohort due to insufficient HbA1c records to assess good/poor GC. Additionally, 2298 patients were excluded, including 1733 patients without ≥365 days of encounter records in the follow‐up period, three patients died within 365 days after T2DM was identified, and 562 patients with encounter records limited to ED visits or inpatient stays. A total of 19 685 patients with T2DM were included in the final cohort.

### 3.2 Baseline characteristics

The cohort baseline characteristics are presented in Table 1. Subjects in the cohort had an average follow‐up period of 6.36 ± 3.71 years, with 49.99% being males. There were 68.22% of subjects <65 years old, contributing to a mean age of 57.99 ± 13.94. Most subjects were White (79.89%) and non‐Hispanic (91.51%). There were 90.28% of subjects with a BMI ≥25 kg/m^2^, including 21.32% overweight, 46.80% obese, and 22.16% severely obese. While 57.28% of subjects were nonsmokers, 11.63% and 18.43% were current smokers and former smokers, respectively. There were 63.02% and 72.66% of subjects in good baseline SBP and DBP control, respectively. Besides, 84.48% of subjects had good baseline LDL control. There were 71.72% of subjects without T2DM complications at baseline and 22.00% with one complication. In addition, 82.92% of patients had 1–2 CCI at baseline. About 73% of patients had a single GLM class at baseline, while 27.04% had ≥2. Metformin was the most prescribed (65.97%) in all GLM classes. About 27% of patients had sulfonylureas, making sulfonylureas the second common baseline GLM class. Our cohort had a mean baseline HbA1c of 7.49 ± 1.62%, and the percentage of good baseline GC was 56.50%. The mean HVS was 0.42 ± 0.34%, with 33.31% of subjects having HVS 0–20. In our study cohort, 5431 experienced MACE, including 4453 stroke cases, 977 AMI cases, and one death.

**TABLE 1:** Baseline cohort characteristics.

	Total subjects = 19 685	
	Numbers/means	Percentage/SD
Years of follow‐up	6.36	3.71
Sex		
Male	9845	49.99%
Female	9840	50.01%
Age (years)	57.99	13.94
Age groups		
<65	13 430	68.22%
≥65	6255	31.78%
Race		
White	15 726	79.89%
Black or African American	1625	8.26%
Asian	540	2.74%
Multiple races	327	1.66%
American Indian or Alaska Native	46	0.23%
Native Hawaiian or Other	14	0.07%
Unidentified	1407	7.15%
Ethnicity		
Hispanic	1137	5.78%
Non‐Hispanic	18 014	91.51%
Unidentified	534	2.72%
BMI (kg/m^2^)	34.95	8.49
BMI Groups^a^		
Underweight	68	0.35%
Normal	1589	8.07%
Overweight	4197	21.32%
Obese	9212	46.80%
Severely obese	4362	22.16%
Unidentified	257	1.31%
Smoking		
Smokers	2290	11.63%
Former smokers	3627	18.43%
Nonsmokers	11 275	57.28%
Unidentified	2493	12.66%
Complications (numbers)		
0	14 118	71.72%
1	4330	22.00%
2	855	4.34%
≥3	382	1.94%
Comorbidities (CCI scores)		
1–2	16 323	82.92%
3–4	2548	12.94%
≥5	814	4.14%
MACE		
No	14 254	72.41%
Yes	5431	27.59%
Stroke	4453	81.99%
AMI	977	17.99%
Death	1	0.02%
SBP control		
Good	12 405	63.02%
Poor	7280	36.98%
DBP control		
Good	14 303	72.66%
Poor	5382	27.34%
LDL control		
Good	16 630	84.48%
Poor	3055	15.52%
HbA1c (%)	7.49	1.62
Glycemic control		
Good	11 123	56.50%
Poor	8562	43.50%
HVS	0.42	0.34
HVS groups		
0–20	6558	33.31%
21–40	3477	17.66%
41–60	3973	20.18%
61–80	2487	12.63%
81–100	3190	16.22%
GLM class^b^ (counts)		
1	14 362	72.96%
2	4104	20.85%
≥3	1219	6.19%
GLMs		
Metformin	12 987	65.97%
Sulfonylureas	5280	26.82%
Thiazolidinediones	1804	9.16%
DPP‐4 inhibitors	2381	12.10%
GLP‐1 RAs	1328	6.75%
SGLT‐2 inhibitors	1153	5.86%
Insulin	1067	5.42%
Other GLMs	391	1.99%

Abbreviations: BMI, body mass index; CCI, Charlson comorbidity index; DBP, diastolic blood pressure; DPP‐4 inhibitor, dipeptidyl peptidase‐4 inhibitors; GLM, glucose‐lowering medication; GLP‐1 RAs, glucagon‐like peptide‐1 receptor agonists; HVS, HbA1c variability score; HbA1c, Hemoglobin A1c; LDL, low‐density lipoprotein; MACE, major adverse cardiovascular event; SBP, systolic blood pressure; SD, standard deviation; SGLT‐2 inhibitors, sodium‐glucose cotransporter 2 inhibitors.

^a^ Underweight: BMI < 18.5 kg/m^2^; normal: 18.5–24.9 kg/m^2^; overweight: 25–29.9 kg/m^2^; obese: 30–39.9 kg/m^2^; and severely obese: BMI≥40 kg/m^2^.

^b^ GLM class was the counts of different GLM used at baseline, including biguanides (metformin), sulfonylureas, thiazolidinediones, GLP‐1 RAs, SGLT‐2 inhibitors, insulin, and so forth.

### 3.3 Statistical analysis

#### 3.3.1 Cox regression

**FIGURE 1:**
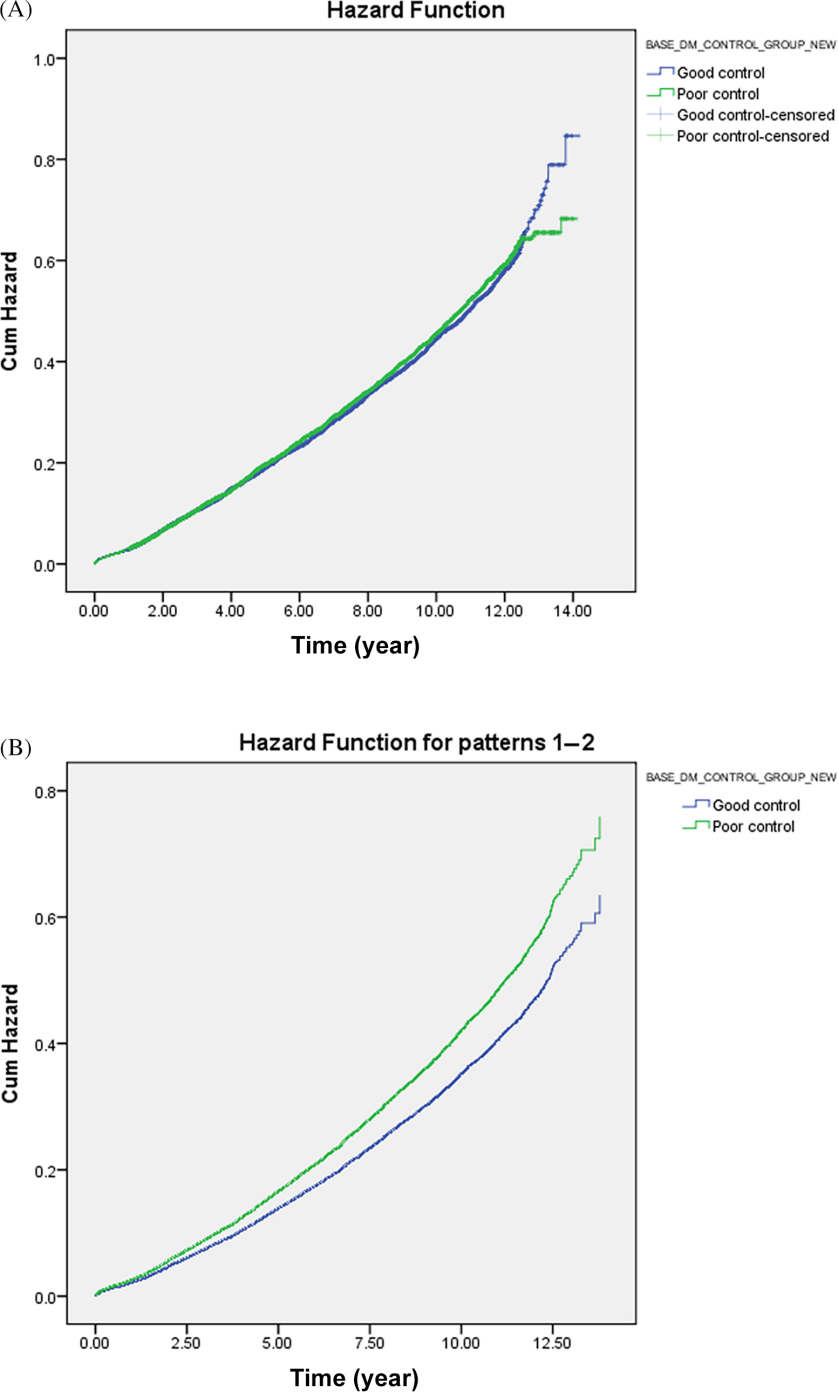
(A) KM survival analysis for MACE risk difference between subjects with good/poor baseline glycemic control. Log‐rank test significance = 0.520. (B) Multivariate Cox regression for MACE risk difference between subjects with good/poor baseline glycemic control. KM, Kaplan–Meier; MACE, major adverse cardiovascular event.

The result of univariate Cox regression is presented in Table S2. For the multivariate Cox regression (Table 2), females had 0.813 (CI: 0.767–0.863) times lower MACE risk than males; being ≥65 years of age had 2.640 (CI: 2.473–2.871) times higher MACE risk than those <65 of age. Significance could be found in the overweight, obese, and severely obese groups, with BMI >25 kg/m^2^ indicating a lower MACE risk. While no significance was found between those with normal BMI and those underweight, being underweight had a 1.432 (CI: 0.915–2.212) times higher MACE risk. Current smokers and former smokers had 1.413 (CI: 1.285–1.555) and 1.205 (CI: 1.117–1.301) times higher MACE risk than nonsmokers. MACE risk increased as the number of existing baseline complications or CCI scores increased. Those with good baseline SBP and DBP control had an MACE risk of 0.732 (CI: 0.686–0.781) and 0.803 (CI: 0.742–0.870), respectively. Good LDL control reduced a MACE risk of 0.873 (CI: 0.799–0.954). Figure 1A,B shows the KM survival curve and multivariate Cox regression of good/poor baseline GC. Subjects in good baseline control had 0.837 (CI: 0.782–0.895) times lower MACE risk. Having >1 GLM class at baseline was associated with higher MACE risk. Those only with one GLM class at baseline were 0.396 (CI: 0.224–0.702) times less likely to experience MACE. No significant MACE risk difference was found in those with/without GLP‐1 RAs and SGLT‐2 inhibitors at baseline. Relatively lower MACE risk was shown in those without sulfonylureas (HR: 0.595; CI: 0.456–0.777) and insulin use at baseline (HR: 0.469; CI: 0.355–0.621).

**TABLE 2:** Multivariate Cox regression for the MACE hazard ratio.

Covariates	HR	95% CI	*p* value
Sex			
Males	Ref.	‐	‐
Females	0.813	0.767–0.863	<0.001***
Age (years)			
<65	Ref.	‐	‐
≥65	2.640	2.473–2.817	<0.001***
Race			<0.001***
White	Ref.	‐	‐
Black or African American	0.809	0.719–0.909	<0.001***
Asian	0.639	0.495–0.825	0.001**
Multiple races	0.649	0.463–0.909	0.012*
American Indian or Alaska Native	0.540	0.242–1.205	0.132
Native Hawaiian or other	0.864	0.322–2.321	0.773
Unidentified	0.678	0.557–0.824	<0.001***
Ethnicity			0.002**
Non‐Hispanic	Ref.	‐	‐
Hispanic	1.029	0.839–1.263	0.783
Unidentified	1.464	1.140–1.880	0.003**
BMI^a^			<0.001***
Normal	Ref.	‐	‐
Underweight	1.432	0.915–2.212	0.117
Overweight	0.871	0.782–0.969	0.011*
Obese	0.758	0.685–0.839	<0.001***
Severely obese	0.735	0.655–0.825	<0.001***
Unidentified	0.811	0.578–1.139	0.227
Smoking			<0.001***
Nonsmokers	Ref.	‐	‐
Smokers	1.413	1.285–1.555	<0.001***
Former smokers	1.205	1.117–1.301	<0.001***
Unidentified	1.453	1.346–1.568	<0.001***
Complications			<0.001***
0	Ref.	‐	‐
1	0.944	0.881–1.012	0.105
2	1.299	1.149–1.470	<0.001***
≥3	1.906	1.599–2.273	<0.001***
Comorbidities (CCI score)			<0.001***
1–2	Ref.	‐	‐
3–4	1.625	1.503–1.756	<0.001***
≥5	2.633	2.332–2.971	<0.001***
SBP control			
Poor	Ref.	‐	‐
Good	0.732	0.686–0.781	<0.001***
DBP control			
Poor	Ref.	‐	‐
Good	0.803	0.742–0.870	<0.001***
LDL control			
Poor	Ref.	‐	‐
Good	0.873	0.799–0.954	0.003**
Glycemic control			
Poor	Ref.	‐	‐
Good	0.837	0.782–0.895	<0.001***
HVS			
80–100	Ref.		
0–20	0.519	0.470–0.573	<0.001***
21–40	0.584	0.530–0.664	<0.001***
41–60	0.640	0.582–0.704	<0.001***
61–80	0.725	0.653–0.804	<0.001***
GLM class (counts)^b^			0.005**
≥3	Ref.	‐	‐
2	0.666	0.509–0.871	0.003**
1	0.396	0.224–0.702	0.002**
GLMs (Without vs. With)			
Metformin	0.704	0.540–0.917	0.009**
Sulfonylureas	0.595	0.456–0.777	<0.001***
Thiazolidinediones	0.669	0.509–0.880	0.006**
DPP‐4 inhibitors	0.708	0.539–0.930	0.013*
GLP‐1 RAs	0.896	0.672–1.196	0.457
SGLT‐2 inhibitors	0.883	0.660–1.181	0.401
Insulin	0.469	0.355–0.621	<0.001***
Other GLMs	0.620	0.459–0.838	0.004**

Abbreviations: BMI, body mass index; CCI, Charlson comorbidity index; CI, confidence interval; DBP, diastolic blood pressure; DPP‐4 inhibitor, dipeptidyl peptidase‐4 inhibitors; GLM, glucose‐lowering medication; GLP‐1 RAs, glucagon‐like peptide‐1 receptor agonists; HR, hazard ratio; HVS, HAb1c variability scores; LDL, low‐density lipoprotein; MACE, major adverse cardiovascular event; Ref, reference; SBP, systolic blood pressure; SD, standard deviation; SGLT‐2 inhibitors, sodium‐glucose cotransporter 2 inhibitors.

^a^ Underweight: BMI < 18.5 kg/m^2^; normal: 18.5–24.9 kg/m^2^; overweight: 25–29.9 kg/m^2^; obese: 30–39.9 kg/m^2^; and severely obese: BMI≥40 kg/m^2^.

^b^ GLM class was the counts of different GLM used at baseline, including biguanides (metformin), sulfonylureas, thiazolidinediones, GLP‐1 RAs, SGLT‐2 inhibitors, insulin, and so forth.

* *p* < 0.05;

** *p* < 0.01;

*** *p* < 0.001.

#### 3.3.2 GC‐stratified HVS and MACE risk

Table 3 presents the result of HVS on MACE risk stratifying by baseline GC, with HVS subgroups referencing from HVS 81–100. All GC‐stratified HVS subgroups reached significance. Good baseline GC subjects with HVS 0–20 had the lowest MACE risk (HR: 0.500; CI: 0.440–0.568). In contrast, poor GC subjects with HVS 61–80 had more increased MACE risk (HR: 0.855; CI: 0.756–0.966). MACE risk increased as HVS increased in both baseline GC statuses.

**TABLE 3:** HVS subgroup analysis for MACE risk, stratified by baseline glycemic control.

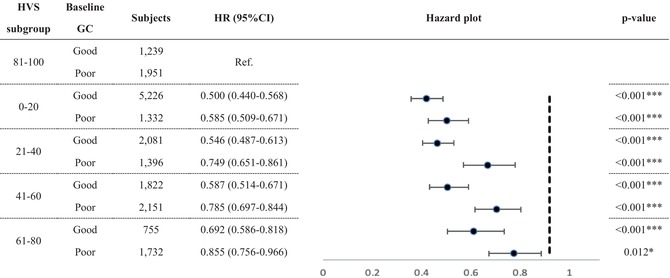

*Note*: HVS 81–100 with good/poor baseline GC were the referenced group for each GC‐stratified HVS subgroup.

Abbreviations: CI, confidence interval; GC, glycemic control; HR, hazard ratio; HVS, HbA1c variability score; HbA1c, Hemoglobin A1c; MACE, major adverse cardiovascular event; Ref, reference.

* *p* < 0.05;

*** *p* < 0.001.

#### 3.3.3 Baseline GLMs and MACE risk

**FIGURE 2:**
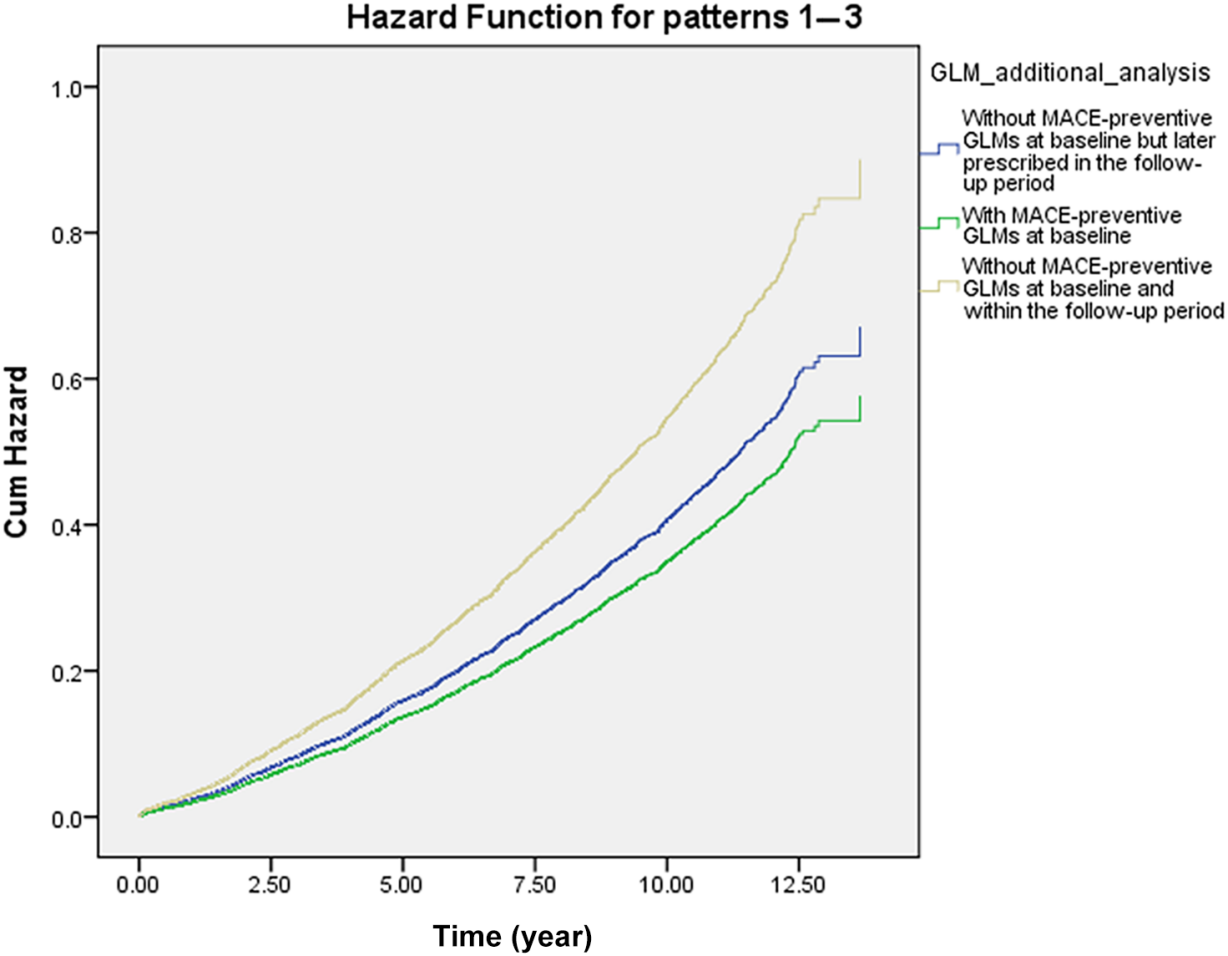
Subgroup analysis for MACE reduction among subjects with poor baseline glycemic control considering the time of MACE‐preventive GLMs being prescribed. GLM, glucose‐lowering medication; MACE, major adverse cardiovascular event.

Table 4 shows the MACE survival difference in the subgroup analysis of baseline GLM use. Among 19 685 patients with T2DM, 13 374 were selected in the subgroup analysis. Compared with those without longitudinal MACE‐preventive GLMs, those with longitudinal MACE‐preventive GLMs showed a 0.681 (CI: 0.635–0.731) times lower MACE risk. While all selected GLMs presented MACE‐preventive effects, the MACE risk reduction effect could range from the lowest, pioglitazone (HR: 0.825; CI: 0.714–0.954), to the highest, semaglutide (HR: 0.439; CI: 0.141–1.364). Subjects with poor baseline GC were selected and further classified according to the prescribed timing of MACE‐preventive GLM. Those without MACE‐preventive GLMs at baseline and in the follow‐up period showed more elevated MACE risk (Figure 2). In comparison, those with MACE‐preventive GLMs at baseline (HR: 0.641; CI: 0.563–0.792) and those without MACE‐preventive GLMs at baseline but later prescribed in the follow‐up period (HR: 0.745; CI: 0.670–0.830) had relatively decreased MACE risk.

**TABLE 4:** Subgroup analysis for glucose‐lowering medications.

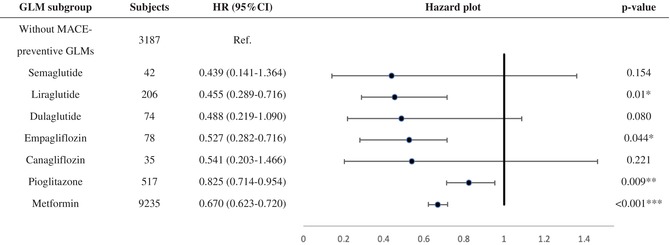

*Note*: Subjects who received a single MACE‐preventive GLM at baseline and constantly received the same prescription for 365 days after T2DM diagnosis were included as the experimental group. In contrast, those without MACE‐preventive GLMs were included as the control group.

Abbreviations: GLM, glucose‐lowering medication; HR, hazard ratio; MACE, major adverse cardiovascular event; Ref, reference.

* *p* < 0.05;

** *p* < 0.01;

*** *p* < 0.001.

## 4 DISCUSSION

Our study not only identified MACE risk factors but also reinforced the role of GC and using MACE‐preventive GLMs in primary MACE prevention. Similar to other studies,^24^, ^25^, ^26^ our study identified good baseline GC as MACE‐preventive in the multivariate Cox regression. KM survival analysis and univariate Cox regression did not recognize the significance of good baseline GC on MACE risk. Insignificance may be attributed to whether covariates were included in the statistical method, which enabled covariate adjustment.^60^, ^61^ The model should include multiple variables to provide an objective outcome comparison and avoid bias, especially when prior studies had identified covariates.^62^ In addition, various risk factors need to be considered concurrently when preventing MACE among patients with T2DM.^24^ Therefore, multivariate Cox regression is more reliable than the other two univariate analyses when interpreting our study outcomes.

As in previous studies,^63^, ^64^ we identified HVS as an essential MACE indicator. Furthermore, our study also found that the MACE risk increased with a higher HVS level, as in two recent studies.^64^, ^65^ Ceriello et al. found that those “not at A1c target” had relatively insignificant MACE risk increases compared with their counterparts. However, our analysis indicated that those with poor baseline GC had more elevated MACE risk than those in good control within the same HVS sector. Besides, our subgroup analysis observed that those with good baseline GC seemed to have higher HVS boundaries, as MACE risk increased from 0.587 in HVS 41–60 to 0.692 in HVS 61–80. In contrast, MACE risk increases from 0.585 in HVS 0–20 to 0.749 in HVS 21–40 among those with poor baseline GC. The results implied that HVS boundaries for MACE risk may differ from good and poor GC. Nevertheless, the correlation needs more research due to the complexity of various MACE determinants.

While studies confirm the general association between obesity and an increased CVD risk,^66^, ^67^ the effect of BMI on CVD risk may differ within the population. Lee et al. observed a significantly lower MACE risk and all‐cause mortality among overweight and obese individuals for 48 438 diabetes patients.^68^ Another prospective study that included 15 785 individuals with stable coronary heart disease also found higher MACE risk among those with BMI <20 kg/m^2^ in the basic and fully adjusted models.^69^ Our study, with elevated BMI cohort characteristics, also observed increased and decreased MACE risk among those with BMI <18.5 and ≥25, respectively. To explain the finding, a study proposed three explanations regarding how elevated BMI may reduce the MACE risk.^70^ GLM use should be concurrently considered when assessing the correlation between BMI and MACE risk in the long run since SGLT‐2 inhibitors and GLP‐1 RAs also have weight loss effects.^28^ For instance, semaglutide is also marketed for obesity, besides T2DM. Therefore, the exact extent to which BMI affects MACE risk by population and the MACE‐preventive effect of using GLMs for patients with T2DM with higher BMI should be further defined.

Our study indicated that those without sulfonylureas (HR: 0.589; CI: 0.459–0.756) and insulin (HR: 0.460; CI: 0.354–0.597) at baseline had lower MACE risk. The study outcomes align with a systemic review that included 126 randomized clinical trials indicating a higher MACE risk among those with insulin and sulfonylurea‐based regimens than the placebo group.^71^ The reason may be sulfonylureas' and insulin's significantly higher hypoglycemia risk.^28^, ^72^ Studies summarized that the rate of severe hypoglycemia is 3.9% patient‐years among those with sulfonylureas,^73^ and those who administer insulin experience 2.5 severe hypoglycemia episodes per person yearly.^74^ Severe hypoglycemia can increase the CVD risk by 2.05.^75^ Additionally, besides AMI, stroke, and heart failure, severe hypoglycemia is also associated with increased all‐cause mortality.^76^ While our study only identified one mortality case, how hypoglycemia can contribute to increased mortality was reported in the ACCORD study.^77^

In the subgroup analysis, semaglutide provided the most significant MACE‐preventive effect (HR: 0.439; CI: 0.141–1.364), while pioglitazone was, relatively, the least effective (HR: 0.825; CI: 0.714–0.954). Notably, imbalanced subject numbers between the subgroups were observed. How first‐line GLM was suggested may explain the imbalanced subject numbers. While prescribers should consider patient characteristics and health conditions when selecting initial antidiabetic treatment,^28^, ^78^ ADA recommended metformin as the initial T2DM pharmacologic therapy before 2022, considering cost, safety, side effects, antihyperglycemic effect, and CVD benefits.^28^, ^78^ Furthermore, SGLT‐2 inhibitors and GLP‐1 RAs are newer GLMs that have gradually been used over the past decade. As a result, our cohort identifying new T2DM subjects from early 2011 may explain why 69.1% of subjects in the subgroup analysis had metformin as the only GLM in the baseline GLM regimen. Although semaglutide, dulaglutide, and canagliflozin could not meet significance, the subgroup analysis potentially revealed their better MACE‐preventive effect in primary MACE prevention for patients with T2DM from real‐world data. As these agents are now to be considered the first‐line GLM used by patients with T2DM with established coronary artery disease, peripheral artery disease, or CVD, the T2DM population may collectively experience further MACE risk reduction.^28^ When focusing on poor baseline GC subjects, the subgroup analysis (Figure 2) presented the benefits of prescribing GLMs in primary MACE prevention for patients with early T2DM duration. Nonetheless, the exact time and duration of MACE‐preventive GLMs should be added and kept, respectively, which requires more studies to reveal.

This study has several limitations. Data discontinuity due to visiting providers outside the same EHR‐using institutions may be an inevitable concern for EHR studies.^43^, ^79^ Besides, this study defined one's T2DM duration according to the EHR data in the PaTH database instead of the actual duration. One possibly had already been diagnosed with T2DM before the index date. Notwithstanding, about 72% of subjects without baseline T2DM complications and 73% only had one GLM class at baseline, indicating the cohort was overall in an early T2DM duration. The study population had an imbalanced race and ethnicity distribution, with 79.8% being White and 91.51% being non‐Hispanic, which limits the generalizability.^80^ PaTH EHR data did not incorporate SDoH information. Studies revealed that SDoH could significantly impact diabetes outcomes^81^, ^82^ and MACE risk.^83^, ^84^ Additionally, the ADA suggested that physical activity and diet are also essential interventions besides GLMs.^85^ Therefore, SDoH and one's physical activity and diet should be included as determinants if possible. Our study only included patients with T2DM using GLMs in order to validate EHR‐based T2DM diagnosis better. Consequently, those in good GC who were under lifestyle intervention without GLMs may be excluded from our study. Last, the subgroup analysis for GLMs only provided the prescription continuity for a year, so alterations to the GLM regimen beyond were not documented.

Three strengths can be observed from this study. (1) T2DM diagnosis in our cohort was based on previous EHR‐based studies with proven effectiveness^39^, ^40^; (2) we only included patients with ≥365 days of encounter records to ensure they were not incident cases without a sufficient follow‐up period. Additionally, patients only with ED visits or inpatient stays were excluded for irregular follow‐up; (3) the study provides robust data selection and processing with up to 11 years of the follow‐up period, establishing a solid longitudinal feature.

## 5 CONCLUSION

This study identified MACE risk factors among patients with T2DM. Early GC control, continuous HbA1c management using HVS, and the use of MACE‐preventive GLMs are crucial for reducing MACE risk in these patients. Future studies should consider other potential determinants of MACE, such as the influence of GLM on HVS, social determinants of health, physical activity, and dietary habits.

## AUTHOR CONTRIBUTIONS

All authors contributed to the study design, data collection and analysis, study outcomes interpretations, and manuscript construction.

## FUNDING INFORMATION

The project described was supported by the National Center for Advancing Translational Sciences, National Institutes of Health, through Grant UL1 TR002014 and Patient‐Centered Outcomes Research Institute through PCORnet Clinical Research Network Infrastructure 1259. The content is solely the responsibility of the authors and does not necessarily represent the official views of the NIH.

## CONFLICT OF INTEREST STATEMENT

The authors declare no conflicts of interest.

## Supporting information


**Data S1.** Supporting Information.
