# Supplementary material for: Major adverse cardiovascular events’ reduction and their association with glucose‐lowering medications and glycemic control among patients with type 2 diabetes: A retrospective cohort study using electronic health records
Source: J Diabetes. 2024 Oct 21;16(10):e13604. doi: 10.1111/1753-0407.13604 (PMC11492400; doi:10.1111/1753-0407.13604)
Supplement: Supplementary file 1 — Data S1. Supporting Information. [file JDB-16-e13604-s001.docx]

**Supplementary**

**Figure S1: The concept of the initial cohort**


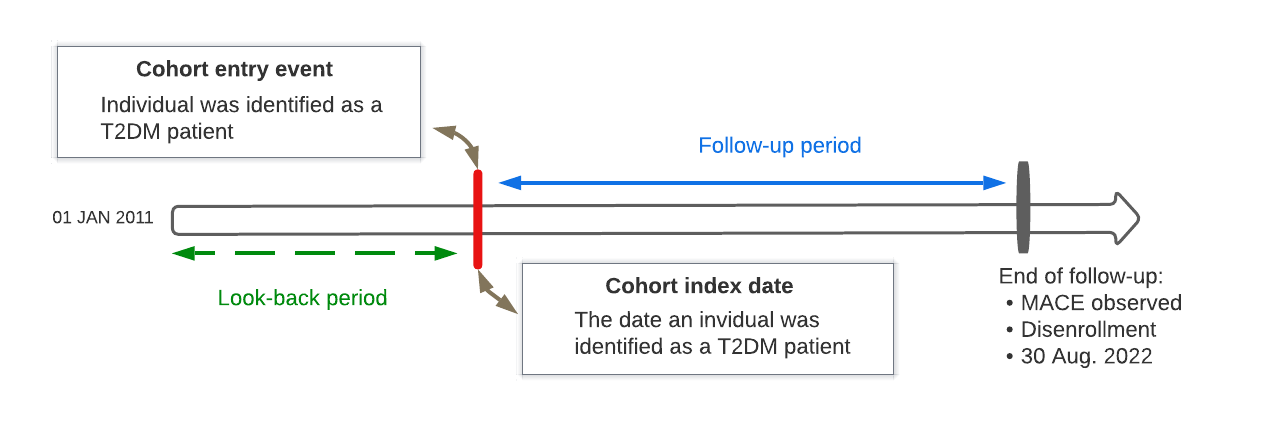


**Abbreviations:** MACE, major adverse cardiovascular event; T2DM, type 2 diabetes

**Figure S2: Cohort selection process**


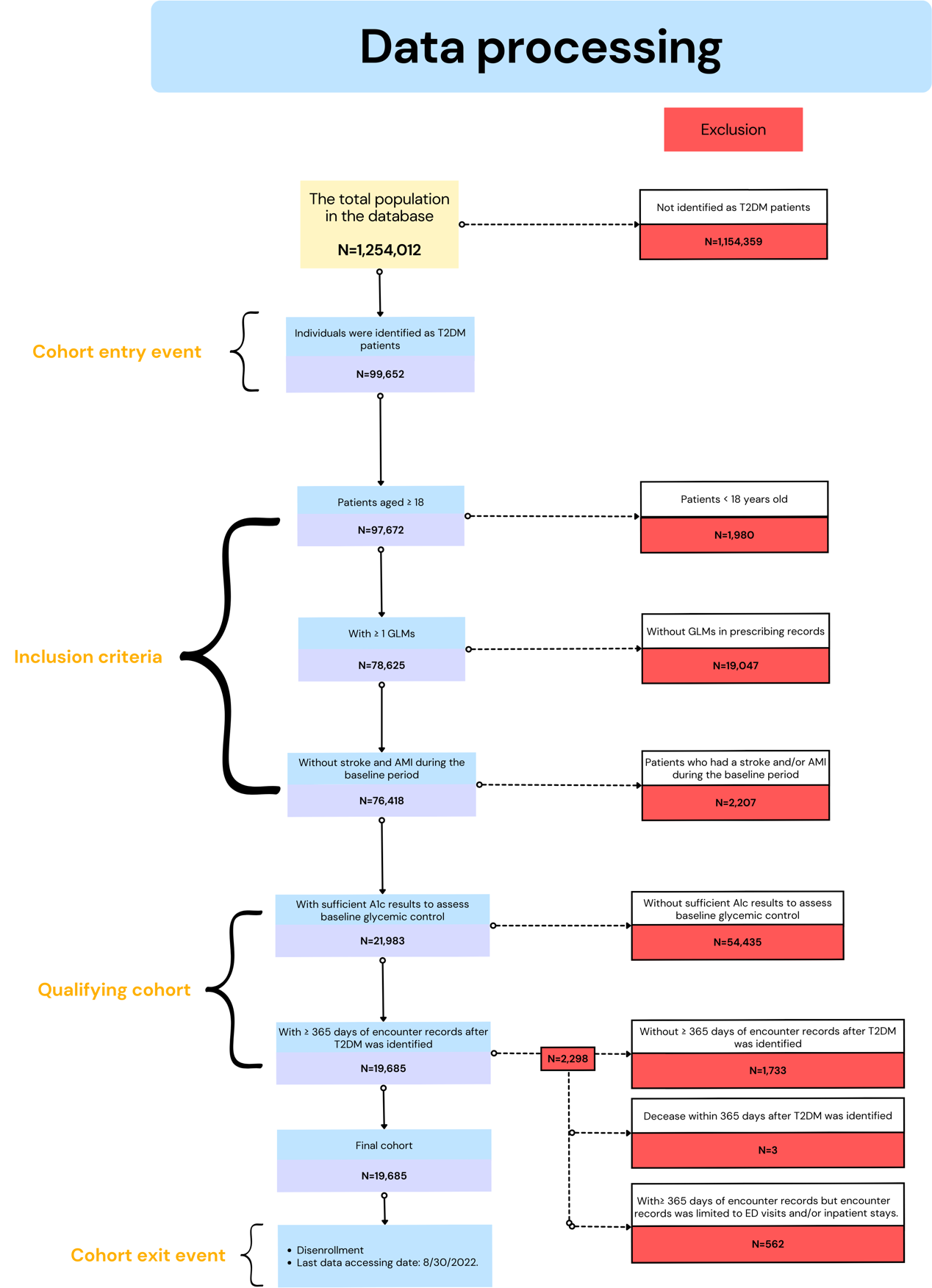


**Abbreviations:** AMI, acute myocardial infarction; ED, emergency department; GLM, glucose-lowering medication; T2DM, type 2 diabetes

**Figure S3: Method of identifying T2DM patients in this cohort**


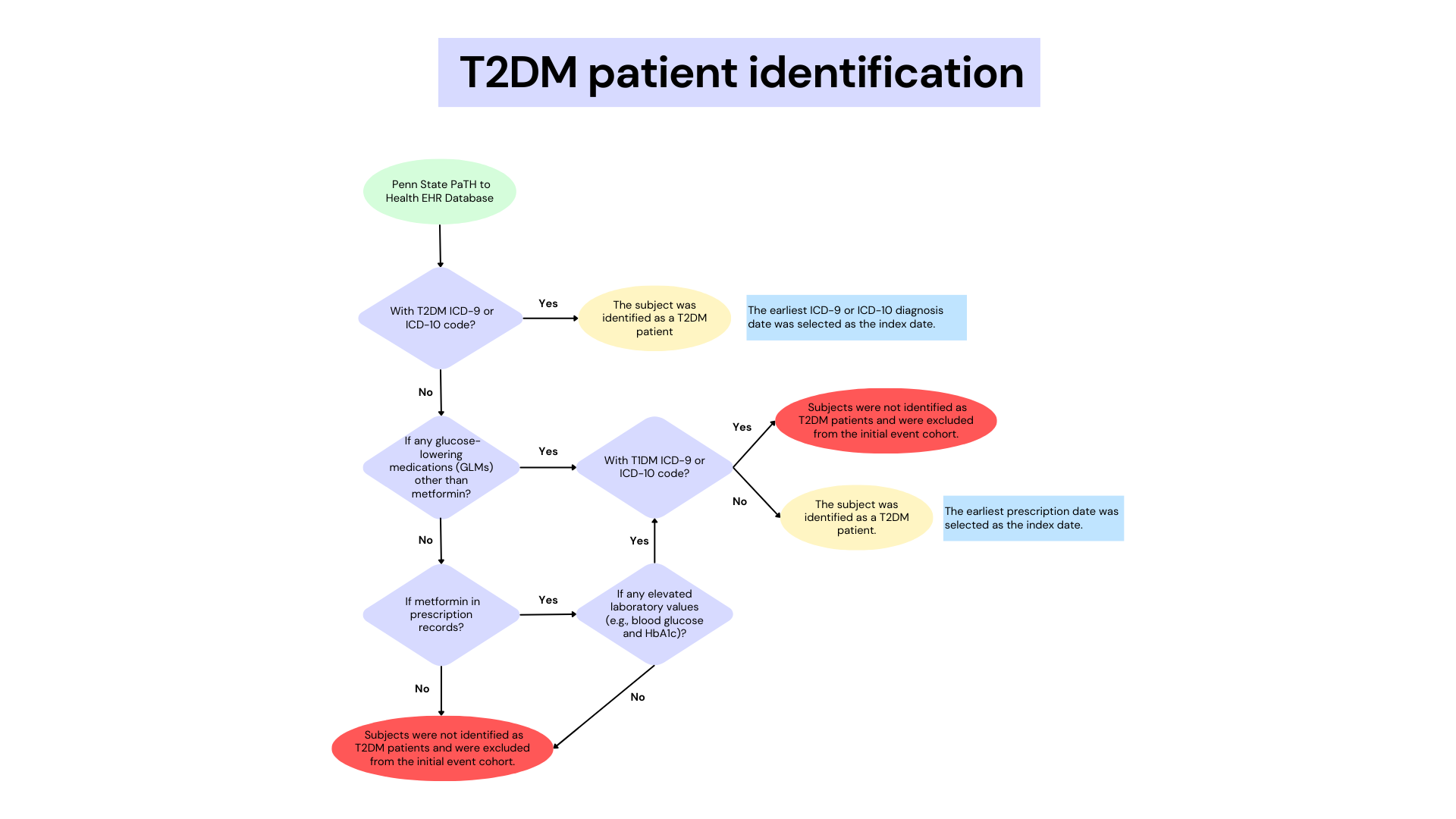


**Abbreviations:** EHR, electronic health record; GLM, glucose-lowering medication; T2DM, type 2 diabetes

**Table S1: Glucose-lowering medications included in this study**

| **Therapeutic Class** | **Generic Name** | **Route of Administration** |
| --- | --- | --- |
| Biguanide | Metformin | Oral |
| Sulfonylureas | Chlorpropamide  Tolazamide  Tolbutamide  Glimepiride  Glipizide  Glyburide | Oral |
| TZD | Pioglitazone  Rosiglitazone | Oral |
| DPP-4 inhibitors | Alogliptin  Linagliptin  Saxagliptin  Sitagliptin | Oral |
| GLP-1 RAs | Exenatide | SubQ |
|  | Lixisenatide |  |
|  | Tirzepatide |  |
|  | Albiglutide |  |
|  | Dulaglutide |  |
|  | Liraglutide |  |
|  | Albiglutide |  |
|  | Semaglutide | SubQ (RYBELSUS^®^ as oral tablet was not included) |
| SGLT-2 inhibitors | Canagliflozin | Oral |
|  | Dapagliflozin |  |
|  | Empagliflozin |  |
|  | Ertugliflozin |  |
| Insulin & analogues | Insulin aspart | SubQ  (Afrezza® is the only oral inhaled) |
|  | Insulin glulisine |  |
|  | Insulin lispro |  |
|  | Regular |  |
|  | NPH |  |
|  | Insulin detemir |  |
|  | Insulin glargine |  |
| Others | Repaglinide | Oral |
|  | Nateglinide |  |
|  | Miglitol |  |
|  | Acarbose |  |
|  | Pramlintide | SubQ |

**Note:** Glucose-lowering medications used by the subjects included as a single agent listed above or came in combination.

**Abbreviations:** DPP-4 inhibitor, dipeptidyl peptidase-4 inhibitors; GLP-1 RAs, glucagon-like peptide-1 receptor agonists; SGLT-2 inhibitors, sodium-glucose cotransporter 2 inhibitors

**Table S2: Univariate Cox regression for the hazard ratio of MACE**

| **Vavirables** | **HR** | **95% CI** | **p-value** |
| --- | --- | --- | --- |
| **Sex**  (Female v.s. Males) | 0.747 | 0.708 – 0.788 | <0.001*** |
| **Age**  (≥65y v.s. <65y) | 2.674 | 2.535 – 2.820 | <0.001*** |
| **Race** | | |  |
| White | Ref. | - | - |
| Black or African American | 0.725 | 0.650 – 0.809 | <0.001*** |
| Asian | 0.572 | 0.449 – 0.730 | <0.001*** |
| Multiple races | 0.651 | 0.499 – 0.849 | 0.002** |
| American Indian or Alaska Native | 0.469 | 0.211 – 1.045 | 0.064 |
| Native Hawaiian or other | 1.509 | 0.628 – 3.628 | 0.358 |
| Unidentified | 0.588 | 0.512 – 0.675 | <0.001*** |
| **Ethnicity** | | |  |
| Non-Hispanic | Ref. | - | - |
| Hispanic | 0.690 | 0.597 – 0.797 | <0.001*** |
| Unidentified | 1.036 | 0.853 – 1.258 | 0.720 |
| **BMI**^†^ | | |  |
| Normal | Ref. | - | - |
| Underweight | 1.236 | 0.821 – 1.860 | 0.310 |
| Overweight | 0.871 | 0.788 – 0.962 | 0.006** |
| Obese | 0.680 | 0.620 – 0.746 | <0.001*** |
| Severely obese | 0.576 | 0.519 – 0.639 | <0.001*** |
| Unidentified | 0.702 | 0.513 – 0.961 | 0.027* |
| **Smoking** | | |  |
| Non-smokers | Ref. | - | - |
| Smokers | 1.219 | 1.115 – 1.332 | <0.001*** |
| Former smokers | 1.379 | 1.285 – 1.480 | <0.001*** |
| Unidentified | 1.675 | 1.562 – 1.796 | <0.001*** |
| **Complications** | | |  |
| 0 | Ref. | - | - |
| 1 | 1.052 | 0.987 – 1.121 | 0.117 |
| 2 | 1.714 | 1.531 – 1.918 | <0.001*** |
| ≥3 | 2.480 | 2.116 – 2.908 | <0.001*** |
| **Comorbidities (CCI Score)** | | |  |
| 1-2 CCI | Ref. | - | - |
| 3-4 CCI | 1.911 | 1.779 – 2.052 | <0.001*** |
| ≥5 | 2.880 | 2.575 – 3.221 | <0.001*** |
| **SBP control**  (Good v.s. Poor) | 0.729 | 0.690 – 0.770 | <0.001*** |
| **DBP control**  (Good v.s. Poor) | 0.754 | 0.704 – 0.807 | <0.001*** |
| **LDL control**  (Good v.s. Poor) | 0.904 | 0.837– 0.975 | 0.009** |
| **Glycemic control**  (Good v.s. Poor) | 0.983 | 0.932 – 1.036 | 0.520 |
| **HVS** |  |  |  |
| 80-100 | Ref. |  |  |
| 0-20 | 0.608 | 0.626 – 0.734 | <0.001*** |
| 21-40 | 0.678 | 0.556 – 0.664 | <0.001*** |
| 41-60 | 0.703 | 0.645 – 0.766 | <0.001*** |
| 61-80 | 0.729 | 0.663 – 0.803 | <0.001*** |
| **GLM class (counts)**^‡^ | | |  |
| ≥ 3 baseline GLM classes | Ref. | - | - |
| Two baseline GLM classes | 0.944 | 0.884 – 1.008 | 0.086 |
| One baseline GLM class | 0.772 | 0.656 – 0.868 | <0.001*** |
| **GLMs (Without v.s. With)** | | | |
| Metformin | 1.244 | 1.179 – 1.313 | <0.001*** |
| Sulfonylureas | 0.831 | 0.785 – 0.879 | <0.001*** |
| Thiazolidinediones | 1.005 | 0.925 – 1.091 | 0.909 |
| DPP-4 inhibitors | 1.079 | 0.995 – 1.170 | 0.066 |
| GLP-1 RAs | 1.645 | 1.436 – 1.883 | <0.001*** |
| SGLT-2 inhibitors | 1.588 | 1.377 – 1.832 | <0.001*** |
| Insulin | 0.615 | 0.557 – 0.678 | <0.001*** |
| Other GLM | 0.877 | 0.742 – 1.036 | 0.122 |

**Notes:** †Underweight: BMI<18.5 kg/m^2^; Normal: 18.5-24.9 kg/m^2^; Overweight: 25-29.9 kg/m^2^; Obese: 30-39.9 kg/m^2^; Severely obese: BMI≥40 kg/m^2^. ‡GLM class was the counts of different GLM used at baseline, including biguanides (metformin), sulfonylureas, thiazolidinediones, GLP-1 RAs, SGLT-2 inhibitors, insulin, etc.

**Abbreviations:** BMI, body mass index; CCI, Charlson comorbidity index; CI, confidence interval; DBP, diastolic blood pressure; DPP-4 inhibitor, dipeptidyl peptidase-4 inhibitors; GLM, glucose-lowering medication; GLP-1 RAs, glucagon-like peptide-1 receptor agonists; HR, hazard ratio; HVS, HbA1c variability scores; LDL, low-density lipoprotein; MACE, major adverse cardiovascular event; Ref, reference; SBP, systolic blood pressure; SD, standard deviation; SGLT-2 inhibitors, sodium-glucose cotransporter 2 inhibitors

*p< .05; **p< .01; ***p< .001
